# Comparison of motorcycle taxi driver’s respiratory health using an air quality standard for carbon monoxide in ambient air: a pilot survey in Benin

**DOI:** 10.11604/pamj.2018.30.113.14975

**Published:** 2018-06-12

**Authors:** Herve Lawin, Lucie Ayi Fanou, Arsene Amadohoue Kpangon, Antoine Vikkey Hinson, John Balmes, Jacqueline Wanjiku, Boni Maxime Ale, Benjamin Fayomi

**Affiliations:** 1 Unit of Teaching and Research in Occupational and Environmental Health, Faculty of Health Sciences, University of Abomey-Calavi, Benin; 2 EcoHealth Chair, Faculty of Health Sciences, University of Abomey Calavi, Cotonou, Benin; 3 Laboratoire de Biochimie et de Biologie Moléculaire, FAST/UAC, Bénin; 4 Department of Internal Medicine, University Hospital of Parakou, Benin; 5 Division of Environmental Health Sciences, School of Public Health, University of California, Berkeley, USA; 6 Department of Internal Medicine, Egerton University, Kenya; 7 Institute of Tropical and Infectious diseases, University of Nairobi, Kenya

**Keywords:** Occupational exposure, respiratory symptoms, lung function, carbon monoxide, air quality standards

## Abstract

**Introduction:**

Ambient air quality standards are not designed to protect people occupationally exposed to outdoor air pollution on a routine basis. This study aimed to assess the effect of exceeding the US ambient air quality standard for carbon monoxide (CO) on motorcycle taxi drivers respiratory health.

**Methods:**

A cross-sectional study of 85 current motorcycle taxi drivers with at least 5 years of job tenure in Cotonou (Benin) was conducted. Personal CO was measured with a portable CO data logger for 8 hours per day during working hours. A questionnaire on respiratory symptoms was administered to participants and spirometry was performed. Participants were divided into two groups, those with exposure to CO >9 ppm and ≤9 ppm, according to the US Environmental Protection Agency (EPA) National Ambient Air Quality Standard which is an 8-hour average of 9ppm. 8 and 10 ppm were also used an exposure limit. Analysis was done using these two groups.

**Results:**

Socio-demographic characteristics were well balanced between the two study groups. The drivers with a CO exposure of more than 9ppm had non-significantly more respiratory symptoms (OR=1.67; 95%CI:0.26,10.74), lower FVC and FEV1 compared to the less exposed group but they have a significant lower PEF (-10%, p=0.02). When we used an exposure limit of 8 or 10 ppm the results were not statistically different.

**Conclusion:**

Drivers with a CO exposure >9 ppm tend to have more respiratory problems. More research is needed to reinforce this result in order to improve air quality standards to protect workers occupationally exposed to outdoor air pollution.

## Introduction

In most cities of developing countries, traffic jams and congestion have become routine. Ambient air pollution in these cities has already reached high levels, exceeding the recommended limits set by the World Health Organization (WHO). According to the most recent WHO assessment, 2 million people die prematurely every year due to air pollution with more than half of these cases in developing countries [1]. Traffic-related air pollution is a complex mixture of pollutants derived from many sources and with different spatial distributions [2-5]. There is evidence that people who work in jobs involving road transport may have high exposures to traffic-related air pollution [6]. In many cities in low and middle-income countries (LMIC), especially in Africa, motorcycle taxis are the main mode of local transportation and a relatively large number of people are employed as drivers [7]. Very little is known about the occupational exposure to carbon monoxide (CO) of motorcycle taxi drivers, but it is likely that they experience particularly high exposures because they are in the middle of traffic for much of their work shift. Cotonou, the economic capital of Benin (west Africa) is a highly polluted urban area in Africa [8, 9]. In Benin, motorcycle taxi driving is a major occupation with almost 2.5% of the total population employed in this activity in 2002 [10]. A cross-sectional study conducted among 400 motorcycle taxi drivers, after analysis of the ambient air collected at intersections and motorcycle taxi parks, showed peak concentrations of CO of 38.6 ppm (parts per million) in the morning and 78.6 ppm in the afternoon [8] although national air quality in Benin for CO is 32 ppm (1 hour- average exposure) [11]. A different study carried out in Benin using self-report questionnaires distributed to motorcycle taxi drivers, showed that 23% of the drivers had difficulty breathing [12]. But very little is known about the relationship between exceedances of air quality standards for carbon monoxide and respiratory health disorders among motorcycle taxi drivers. Indeed in Benin, like in most of the countries where there are many motorcycle taxi drivers and other outdoor workers, national air quality standards are not designed to protect workers occupationally exposed to traffic related air pollution (TRAP) in urban areas. In conventional "workplace" (plants/companies) it exist some standards for CO exposure. The American Conference of Governmental Industrial Hygienists sets this level to 25 ppm as an 8-hour average for an 8-hour workday over a 40-hour work week [13] and in Canada it is 35 ppm [14]. Our study aimed to assess respiratory symptoms and lung function of motorcycle taxi drivers exposed to CO above and below the US Environmental Protection Agency (EPA) 8-hours/day ambient air quality standard [15]. This exposure limit is one the most restrictive and is 9 ppm for CO. CO may not have direct effect on respiratory health but has been used in this study as a proxy of air pollution. The study goal was to raise awareness on the need of specific air quality standards for outdoor workers occupationally exposed to TRAP. We hypothesize that that if drivers already have respiratory problems after 8 hours of exposure to CO from TRAP that has exceeded 9 ppm, we may need a CO exposure limit value lower than 9 ppm to protect them from adverse effect of exposure to CO in ambient air.

## Methods

**Study design**: We conducted a cross-sectional study involving 85 current motorcycle taxi drivers in Cotonou, which is the capital city of the Republic of Benin. The study was approved by the ethics and research committee of the "Institut des Sciences Biomédicales Appliquées". All participants provided written informed consent.

**Participants of the study**: The recruitment plan has been extensively described elsewhere [16]. Briefly we systematically sampled every third individual from a list of 300 current male motorcycle taxi drivers with at least 5 years of work experience provided by the main motorcycle taxi driver association in Cotonou and attempted to recruit them into the study. 100 drivers were met but 95 were recruited; 5 participants did not consent. Of the 95 enrolled, only 85 completed the study and 10 were unable to provide valid spirometry.

### Variables and measurement

**Questionnaires**: All participants completed a questionnaire regarding respiratory symptoms (cough, dyspnea, expectorations, chest wheezing) smoking history, socio-demographic characteristics (income per day, number of years attended school, marital status), co-morbidities (previous lung/heart diseases, diabetes, stroke, tuberculosis). The questions on respiratory symptoms were taken from the Burden of Lung Disease Study (BOLD) core questionnaire [16]. The questionnaire was translated and back translated from the local language to French. It was administered by trained staff and data was electronically collected [17]. For the analysis all the respiratory symptoms have been combined to single a variable we named "any respiratory symptoms"; we did the same combination for the co-morbidities and the variable name was "any co-morbidities".

**CO exposure**: It was measured with a portable pre calibrated CO data logger with a USB interface (El-USB-CO^®^, Lascar Electronics, Whiteparish Salisbury, UK). All participants carried the device for 8 hours per day during working hours with a sampling rate of 5 minutes. The device was fixed with a rope to their neck as close as feasible to their breathing zone. They were given the device before starting their working day. The data collected during the 8-hour shift were then uploaded to a computer and the Time Weighted Average (TWA) CO exposure was recorded.

**Spirometry**: Spirometry was performed at the beginning of the study by each participant in the morning hours between 0900-1300 hours before CO exposure monitoring was begun. The study participants completed spirometry according to the American Thoracic Society and European Respiratory Society guidelines [18] with an EasyOne^®^ Spirometer ( ndd, Switzerland) calibrated daily with a 3-L syringe. The spirometric procedure included at least 3 acceptable and repeatable forced vital capacity maneuvers. All the tests were reviewed by an external investigator and the best value was selected for analysis. We reported the forced expiratory volume in one second (FEV1), the forced vital capacity (FVC), and the peak expiratory flow (PEF).

**Data analysis**: The study participants were analyzed in two groups divided according to the US Environmental Protect Agency (US EPA) health-based national air quality standard for CO of 9 parts per million (ppm) measured as an annual second-maximum 8-hour average concentration [15]. We compared characteristics of the two groups of motorcycle taxi drivers using t-test for continuous data and chi square for categorical data. We also performed regression analysis of the association between the TWA CO exposure and lung function parameters. Separate models were fitted with and without adjustment for age, body mass index, year attended school, tobacco smoking, marital status, biomass fuel exposure, income per day and any co-morbidities. We have also changed the exposure limit to 8 ppm and 10 ppm to see how the results varied. All analyses were performed using SPSS 16.0 and a p value <0.05 was significant.

## Results

**Characteristics of the study population**: The characteristics of our study population are reported in Table 1 below. The two groups had almost the same age (43±8 vs. 43±7, p=0.92). The drivers with higher exposure to CO reported less years of driving experience, co-morbidities, education and tobacco exposure than the less exposed group.

**Table 1:** Study population characteristics

	Carbon monoxyde (CO) level		P value
	0-9 ppm (n=64)	> 9ppm (n=21)	
Age (years)^1^	43±8	43±7	0.92
Job duration (years)^1^	13±7	11±4	0.09
BMI (Kg.m-2)^1^	28±9	32±6	0.57
years of education^1^	7±4	5±3	0.06
incomes per day (XOF)^2^	3680±910	3980±785	0.16
Any co-morbidity (%)	24	14	0.54
Exposure to tobacco smoke (%)	36	24	0.43

1 Expressed as mean±standard deviation

2 XOF=local currency in Benin

**Associations between carbon monoxide level in ambient air and respiratory symptoms**: The drivers with a CO exposure of >9ppm had more respiratory symptoms compared to the drivers with CO levels of ≤9ppm; however, this finding was not statistically significant (Table 2). When we used an exposure limit of 8 or 10 ppm the results were not statistically different (results not shown).

**Table 2:** Associations between carbon monoxide level and respiratory symptoms

Any respiratory symptoms						
CO		**Yes**	**No**	**COR^1^**	**AOR^2^**	**95%CI**
	0-9ppm	7	57			
	>9ppm	3	18	1.36	1.67	[0.26, 10.74]

1 Crude odds ratio

2 Adjusted odds ratio

Adjusted for age, body mass index, year attended school, environmental tobacco smoke exposure, and marital status, and biomass fuel exposure, income per day and co-morbidities

**Associations between carbon monoxide level in ambient air and lung function**: The PEF was significantly lower among the motorcycle taxi drivers whose TWA CO exposure exceeded the US EPA air quality standard. These drivers also had lower FVC and FEV1 compared to the less exposed group, however, the differences were not statistically significant (Table 3). When we used an exposure limit of 8 or 10 ppm none of the results were statistically different (results not shown).

**Table 3:** Associations between carbon monoxide level in ambient air and lung function

	CO	Mean±Standard deviation	P value
FEV1(percent predicted^1^)	0-9ppm	91±17	0.70
	>9ppm	89±15	
FVC(percent predicted^1^)	0-9ppm	92±13	0.66
	>9ppm	90±15	
PEF(percent predicted^1^)	0-9ppm	101±27	0.02
	>9ppm	89±18	

1 NHANES predicted values for African Americans

## Discussion

This study compared two groups of motorcycle taxi drivers stratified according to the US EPA's health-based air quality standard which is an 8-hour average of 9ppm. The drivers with a TWA CO exposure >9ppm had more respiratory symptoms and lower lung function, but the only statistically significant difference was for peak expiratory flow (PEF). To the best of our knowledge, this is the first study to report a lung function difference based on CO exposure in this occupational group which includes many workers in African and other low and middle-income countries. A previous study was done in Porto-Novo, Benin [12] with an aim to assess potential respiratory problems among 48 motorcycle taxi drivers compared to a control group composed of people who occasionally practiced sports for health maintenance. A questionnaire, spirometry and 6-minute walk tests were used to identify symptoms and changes in physiological variables that reveal the existence of bronchospasm. The frequency of respiratory symptoms noted among motorcycle taxi drivers was higher than the one recorded among members of the control group. The authors also observed that motorcycle drivers both at rest and after physical effort had significantly lower lung function than those recorded in the control group [12]. Because of the lack of a cabin, exposure to traffic-related air pollution on a motorcycle is probably higher than in an automobile, especially if the windows are closed with air conditioning on in the latter. Studies have so far reported this for PM, CO, NO2 and VOCs [19-23]. This is likely to be because motorcyclists are situated directly in the "stream of pollutants" without any shielding, along with their relatively close proximity to the exhaust tailpipes of traffic ahead. Like many other air pollutants, CO levels in urban regions are highly influenced by such factors as traffic density, traffic congestion and meteorological conditions. Ambient CO concentrations have daily and seasonal variations, as well as complex spatial distributions.

This current US EPA's cut off may be considered in the definition of the specific exposure limit value in this occupational group. Countries have air quality standards for the general population regarding exposure to air pollutants but nothing which takes into account the specificity of the group occupationally exposed like motorcycle taxi drivers, traffic policemen, street and roadside vendors most of the time. Due to the physical exertion and specificity related to these activities, air quality standards may be more restrictive than that of the general population regarding exposure to carbon monoxide in urban area. To the best of our knowledge, there is no international air quality standard for group occupationally exposed to traffic related air pollutants in urban area. The permissible exposure limit for CO in occupational settings recommended by the American Conference of Governmental Industrial Hygienists is 25 ppm as an 8-hour average for an 8-hour workday over a 40-hour workweek [13]. In Canada the weighted average exposure values of CO (for a period of 8 hours/day, 40 hours/week) in the workplace is 35 ppm [14]. If this notion of "occupational settings" or "workplace" includes urban areas polluted by motor vehicle emissions, the results of this current study suggest that this cut off may be reviewed. Indeed we measured the CO for 8 hours during the working day and with a cut off of 9 ppm there is already a trend to more respiratory symptoms and decreased lung function. Potential limitations do exist in our use of personal CO monitoring. CO is a component of emissions from all combustion sources, including burning of biomass and our measurements may have reflected exposures other than from motor vehicle emissions. We also acknowledge that the health effects observed may have been due to other components in the traffic pollution mixture. There is also an insufficient statistical power but the tendency we have observed in this study is quite sufficient to initiate the discussion. The differences in respiratory symptoms, FEV1 and FVC may have been significant with a larger sample size. Another limitation may be lack of generalizability to cities in other countries due to the specific conditions of traffic conditions and motorcycle taxi driving in Cotonou. Our study design may also misclassify the study participants since one exposure measurement may not resume the common exposure of the motorbike taxi drivers.

## Conclusion

Exceeding the exposure limit value of carbon monoxide of 9 ppm among motorcycle taxi drivers in Cotonou was associated with more respiratory disorders although the differences were only statistically different for the peak expiratory flow. This study raises awareness about the personal exposure risks to traffic-related air pollution of motorcycle taxi drivers and suggests that powered follow up study is needed to help policy makers in making effective preventive public health decisions. Air quality standards may take into account the part of the population occupationally exposed to traffic air pollutants in order to define appropriate exposure limit value.

### What is known about this topic

- Commercial drivers are occupationally exposed to ambient air pollution;
- Ambient air quality standards are not commonly designed to protect those occupationally exposed;
- Carbon monoxide is used as a surrogate of ambient air pollution.

### What this study adds

- Commercial drivers who exceed the limit of 9 ppm exposure to CO tend to have more respiratory symptoms;
- This exposure limit may be considered for occupationally exposed to ambient air pollution.
